# Letter to the Editor Re: Oh J., et al. *Nutrients* 2019, *11*, 343

**DOI:** 10.3390/nu11030667

**Published:** 2019-03-20

**Authors:** Eduardo Hernández-Garduño

**Affiliations:** Dirección de Administración y Desarrollo de Personal, Instituto de Seguridad Social del Estado de México y Municipios (ISSEMyM), Toluca 50080, Mexico; epidemiologist.researcher@gmail.com; Tel.: +52-722-214-2367

To the editor,

The case-control study by Oh J. et al. [1] analyzing the association between nontuberculous mycobacterial pulmonary disease (NTM-PD) and serum A, D, E, and B_12_ vitamin status found that vitamin A deficiency (defined as serum vitamin A < 1.05 μmol/L) was strongly associated with NTM-PD as compared to controls. However, no controls had vitamin A, E, or B_12_ deficiencies. Apparently NTM-PD cases were more likely to have vitamin A deficiency than controls (11 cases or 7.3% vs. 0 controls or 0%, respectively) and also more likely to be underweight (25 cases or 16.7% vs. 4 controls or 2.7%, respectively) in the univariable analysis shown in Tables 1 and 2 (*p*-values < 0.01). The rate of vitamin D deficiency was similar in both groups (*p*-value = 0.908). The lack of controls with vitamin deficiencies (except for vitamin D) and the small number of underweight controls (*n* = 4) represent a major limitation in this study and one wonders how the sample size was calculated.

Table 2 shows estimates from a multivariable logistic regression, but it is not clear whether the odds ratios shown were obtained after adjusting for other covariables related with vitamin A including BMI, albumin, total cholesterol, and alanine aminotransferase. This is important particularly for BMI because it showed the strongest positive correlation coefficient with vitamin A (0.367; Table 3). Therefore, BMI is a major confounding variable in the logistic regression analysis on the association between vitamin A deficiency and NTM-PD.

Due to the fact that in the univariable analysis low BMI was associated with NTM-PD in this study and other studies [2,3] and the fact that there were no controls with vitamin A deficiency, an approach to confirm whether there is a linear relationship between vitamin A concentrations and NTM-PD would be to perform a multivariable analysis restricted to people with BMI ≥ 18.5. Analysis by NTM species would also be important to establish which specific species, if any, are associated with vitamin A blood concentrations. Furthermore, treatment outcome apparently was not analyzed using multivariable analysis, which represents another limitation in this study. Even if no variables were statistically associated with treatment outcome in the univariable analysis, those with *p*-values within the borderline of statistical significance may become significant in a multivariable analysis.
